# A computational drug repositioning method applied to rare diseases: Adrenocortical carcinoma

**DOI:** 10.1038/s41598-020-65658-x

**Published:** 2020-06-01

**Authors:** Maryam Lotfi Shahreza, Nasser Ghadiri, James R. Green

**Affiliations:** 1 Department of Electrical and Computer Engineering, Isfahan University of Technology, Isfahan, 84156-83111 Iran; 2 Department of Computer Engineering, University of Shahreza, Shahreza, 86149-56841 Iran; 3 Department of Systems and Computer Engineering, Carleton University, Ottawa, K1S 5B6 Canada

**Keywords:** Computational models, Data integration

## Abstract

Rare or orphan diseases affect only small populations, thereby limiting the economic incentive for the drug development process, often resulting in a lack of progress towards treatment. Drug repositioning is a promising approach in these cases, due to its low cost. In this approach, one attempts to identify new purposes for existing drugs that have already been developed and approved for use. By applying the process of drug repositioning to identify novel treatments for rare diseases, we can overcome the lack of economic incentives and make concrete progress towards new therapies. Adrenocortical Carcinoma (ACC) is a rare disease with no practical and definitive therapeutic approach. We apply Heter-LP, a new method of drug repositioning, to suggest novel therapeutic avenues for ACC. Our analysis identifies innovative putative drug-disease, drug-target, and disease-target relationships for ACC, which include Cosyntropin (drug) and DHCR7, IGF1R, MC1R, MAP3K3, TOP2A (protein targets). When results are analyzed using all available information, a number of novel predicted associations related to ACC appear to be valid according to current knowledge. We expect the predicted relations will be useful for drug repositioning in ACC since the resulting ranked lists of drugs and protein targets can be used to expedite the necessary clinical processes.

## Introduction

A rare (or orphan) disease afflicts only a small percentage of a population. It is not possible to specify a global number for this percentage, as it varies by year and by region^1^. Although the number of people with any given rare disease is small, due to the high number of these diseases, the number of people suffering from at least one rare disease is relatively high. As more than 7,000 rare diseases have been identified to date, they have affected more than 300 million people worldwide^2^.

Some believe that the first step in the development of a new drug for a disease is to examine repositioning an existing drug for that disease. Only if that stage is unsuccessful should one enter the more costly stages of traditional drug discovery^3^. Repositioning a drug is the use of a drug in the treatment of a disease for which it has not been designed or developed from the outset. This approach is efficient regarding speed and cost since many early stages of the design and production of drugs can be omitted; for example, the drug does not require specific evaluations in terms of safety, pharmacology, and toxicology. An additional benefit is that, in the final stages of production, there is no need to develop new infrastructure for manufacturing. In oncology, given the increased demand for new treatments, drug repositioning can be especially beneficial^4^.

The use of drug repositioning for rare diseases has recently attracted significant interest. Drug companies are often hesitant to apply costly traditional drug development processes to rare diseases for two reasons: 1) the lack of economic incentive due to the small number of people affected by each case, and 2) the lack of information and data about these diseases, their drugs, and corresponding drug targets. Therefore, the use of drug repositioning in the treatment of rare diseases is highly promising. In addition to pharmaceutical companies pursuing drug substitution for rare diseases, many government agencies and research institutions are using drug repositioning to pursue humanitarian goals such as improving the living conditions of patients with rare diseases^2^. Considering the small number of people with any particular rare disease, the study of the causes and ways of treating these diseases may represent a first step in the field of personalized medicine^3^.

There are some notable instances of successful drug repositioning related to rare diseases. For example, nicotinamide has been repositioned for the effective treatment of rare Friedreich’s ataxia (This example and others are discussed at http://www.findacure.org.uk/drug-repurposing-case-studies/). A complete list of repositioned drugs (for rare and common diseases) are presented in the drug repositioning portal (http://www.drugrepurposingportal.com). The FDA also has provided a list of repositioned drugs for rare diseases in the Rare Disease Repurposing Database (RDRD) (http://www.fda.gov/ForIndustry/ DevelopingProductsforRareDiseasesConditions). Although most repurposing examples have been identified mostly by accident, they demonstrate the potential ability in this area. It is self-evident that bioinformatics methods to expedite drug repurposing should be considered and expanded.

Integration of information on diseases, drugs, and how they affect and function in the body, with other existing biological and therapeutic information, can contribute to the treatment of diseases, especially rare diseases. Bioinformatics methods can be useful in this regard, with the ability to identify and predict the potential relationships that exist between diseases and drugs and the factors affecting them. Our research team has recently introduced a computational approach to drug repositioning, called Heter-LP^5^. Heter-LP has been tested and evaluated in repositioning drugs for various diseases, yielding significant results^5^. Here, we examine the effects of Heter-LP in the field of drug repositioning in a rare disease named AdrenoCortical Carcinoma (ACC).

ACC is a rare disease for which few effective treatment options are available. The drugs used in the treatment of this disease are highly toxic, and new therapeutic options for ACC are urgently needed^6^.

What follows is a brief overview of ACC. Heter-LP is then briefly introduced. We then provided a description of the clinical and computational methods proposed for drug repositioning for ACC. The results are presented and evaluated in Section 2. In Section 3, the data necessary for the implementation of Heter-LP and our proposed method are introduced. Finally, in Section 4, we provide conclusions and recommendations for future work.

## Adrenocortical Carcinoma (ACC)

The adrenocortical glands are endocrine glands located at the top of each kidney. These glands produce various hormones, including adrenaline, and aldosterone and cortisol steroids. Each gland has an outer cortex and a medullary portion (http://www.clevelandclinicmeded.com/). This gland has among the highest level of blood supply per gram of tissue, such that up to 60 small veins may enter each gland. The normal functioning of the adrenal gland may be impaired by conditions such as infection, tumor, genetic disorders, and autoimmune diseases, or by side effects of medication. These disorders impact health and cause discomfort either directly (such as through infections or autoimmune diseases), or through changes in hormone production (such as some types of Cushing’s syndrome) (http://www.wikipedia.org).

There are two types of dangerous abnormalities in the adrenal glands, ACC, which is related to the cortex, and malignant medullary pheochromocytoma. Both diseases are considered rare diseases^7^. We here focus on ACC. A detailed description of ACC is presented in the supplementary file, section 1.

In this paper, the purpose is not to investigate the diagnostic methods of ACC; we are focused on applying drug repositioning to identify novel treatment avenues for ACC. Interested readers are referred to^8^ for more information on diagnostic methods. Also, Crona *et al*.^9^ reviewed research on diagnosis approaches and improving clinical care of ACC.

The options for drugs to treat ACC are limited. Among the more widely used drugs is mitotane. In some cases, anti-hormonal drugs are also used. The extent and the effect of mitotane are not definitive. Overall, the efficacy of this drug is low, with 19–34% of cases resulting in a minor response; a complete response is seen only rarely. Proponents of mitotane suggest that even if the tumor size is not reduced, it can control abnormal hormone production and eliminate some symptoms^10^. A brief overview of current therapeutic approaches for ACC, along with a summary of the benefits and disadvantages of each, is presented in the supplementary file, section 2.

Another therapeutic approach to different types of cancers is targeted therapy. In this therapeutic approach, one tries to find proteins that affect the disease in a particular way and then develop drugs that target these proteins to enhance or modify their behavior. Recent studies on the molecular mechanisms of ACC tumor growth has suggested novel potential targets^11^. The most promising protein targets for ACC are “Insulin growth factor (IGF-1,2)”^11,12^, “mTOR“^11,13^, “Steroidogenic factor 1 (SF-1)”^11,13^, “CTNNB1”^11^ described in the supplementary file, section 3.

Understanding of the molecular events and signaling pathways that may be disrupted in a disease, will significantly contribute to improving a range of available diagnostic, prognostic, and treatment approaches. We describe some important molecular events and signaling pathways related to ACC in section 4 of the supplementary file.

## Heter-LP

Many real-world problems can be represented by graphs or networks, where items are represented as nodes and relations as edges between these nodes. The majority of graph-related research has focused on homogeneous networks, where all nodes and edges are of the same type. However, more complex problems require more detailed models which cannot be presented by simple homogeneous networks. Their corresponding networks comprise different kinds of nodes and edges; such networks are known as heterogeneous networks. Algorithms that operate on heterogeneous networks differ from those for homogeneous networks in many respects. One such algorithm is link prediction, where one predicts new relations (edges) between different items (nodes), based on previous known relations and attributes (like the weight of relations and some characteristics of nodes).

We modeled a solution to the drug repositioning problem as a link prediction problem over a heterogeneous network consisting of drugs, diseases, drug targets, and relations between all of them, named Heter-LP^5^. Heter-LP is a semi-supervised machine learning method, based on label propagation over a heterogeneous network. Heter-LP consists of three main steps: data modeling, projection, and label propagation. In data modeling, the required data should be gathered and integrated into one heterogeneous network comprising three homogeneous sub-networks (drugs, diseases, and drug targets) and three heterogeneous sub-networks (drug-disease, drug-target, and disease-target). In the projection phase, the topological similarity of each pair of concepts is computed based on heterogeneous sub-networks (The purpose is to determine the similarity of two nodes based on how they are related to other nodes). These topological similarities are integrated with homogeneous sub-networks, and, finally, our proposed label propagation algorithm is applied. This results in new predicted links between drugs, diseases, and targets.

Heter-LP is an iterative approach, and its convergence to a locally optimal solution is proven in^5^. This algorithm could also be used for different proposes in various domains such as social networks, and any other problems need to be modeled by heterogeneous networks.

## Related work

In this section, we first review clinical approaches for drug repositioning related to ACC. We then discuss computational techniques related to rare diseases. Lastly, we introduce some essential general computational methods for drug repositioning, including the widely used DT-Hybrid approach^14,15^.

### Clinical drug repositioning approaches for ACC

Little research has examined the possibility of drug repositioning for treating ACC; furthermore, these few studies have employed only clinical and experimental approaches. The most important drugs discussed in such researches are: mebendazole (MBZ)^16^, bortezomib, ouabain, methotrexate, pyrimethamine^17^, niclosamide^6^, 16 different antiparalytic drugs includes niclosamide^4^, sunitinib^18^, mitotane, etoposide, doxorubicin and cisplatin^19^. A detailed description is presented in the supplementary file.

Despite the efforts of various organizations, such as the European Network for the Study of Adrenal Tumors (ENSAT), in treating ACC and improving the livelihoods of these patients, the disease is still largely uncontrollable, and practical treatment strategies remain elusive^20^.

### Computational drug repositioning approaches for rare diseases

Today, there is an ever-growing amount of biological data with the potential to benefit many application areas. The use of purely clinical and traditional drug repositioning methods are not able to scale and leverage these growing data. Accurate and efficient computational methods are required to fully utilize available data to present the most promising leads for consideration by biological scientists and to extricate researchers from confusion due to a large amount of data. Over the years, different methods for computational drug repositioning and drug-target interaction prediction have been presented, of which only a small number have been explicitly applied to rare diseases. The most important methods are^1,3,21^, described in the supplementary file.

### General computational drug repositioning methods

It is not possible to review all available computational drug repositioning methods here in detail, due to their wide variety. We have categorized these methods and explored one of the most important categories, network-based methods, in our previous paper^22^. One of the best performance metrics for evaluating drug repositioning methods is the area under the receiver operating characteristic (ROC) curve, or the AUC. A number of methods are able to achieve an AUC of greater than 0.9: Bipartite graph learning^23^, BLM-NII^24^, DT-Hybrid^14^, GIP-RLS^25^, Heter-LP^5^, KRM^26^, LPMIHN^27^, MINProp^27^, NBI^28^, NetLapRls^29^, NN^26^, NRWRH^27^, SITAR^30^ (sorted alphabetically). A brief description of each was presented in^22^. In the present study, our method Heter-LP is compared with DT-Hybrid, the leading contemporary method. DT-Hybrid is largely a recommendation method based on projection, where input data are modeled as a bipartite network (a bipartite network is a graph comprising two separate groups of nodes. Each edge connects one node from one group to a node of the other group, i.e. there is no edge between the nodes of the same group), and the similarity of each group of nodes is computed based on the similarity of other groups and across relations between two groups.

## Results

The purpose of the method is to predict the relations that do not already exist in the input data. Novel predictions associated with ACC, resulting from Heter-LP, are presented in Table 1. The DT-Hybrid algorithm could not predict any novel drug-target and drug-disease relations. Its novel disease-target predictions are presented in Table 2. It should be noted that the predicted relations are weighted by a number in the [0, 1] interval, which is considered as the probability of the existence of the relation. Due to the distribution of weights with large differences between them, we used a threshold to select the more effective weights. We only have considered predicted relations with a weight greater than or equal with 0.005 here. A complete list of these predictions and their corresponding weights are available in the supplementary file, section 7.1. A detailed description is provided in the next subsections.

**Table 1:** Predicted relations associated with adrenocortical carcinoma by Heter-LP (weight > =0.005).

Predicted relation	Entity 1	Entity 2
Drug-target	Spironlactone	hsa:1584
	Flavin adenine dinucleotide	hsa:2230
Drug-disease	Cosyntropin	Adrenocortical carcinoma
Disease-target	Adrenocortical carcinoma	hsa:1717 (DHCR7)
		hsa:3480 (IGF1R)
		hsa:4157 (MC1R)
		hsa:4215 (MAP3K3)
		hsa:7153 (TOP2A)

**Table 2:** Predicted relations associated with adrenocortical carcinoma by DT-Hybrid (weight > =0.005).

Predicted relation	Entity 1	Entity 2
Disease-target	Adrenocortical carcinoma	hsa:6409 (SEN2)
		has:595 (CCND1)

To validate each of the novel predicted relations, the three sub-sections below discuss supporting evidence for each type of novel predicted relationships from Tables 1 and 2. In addition, we discuss the association of these results with previously known signaling pathways of ACC in section 7.2 of the supplementary file.

### Drug-target relations

Among the novel predicted relations by Heter-LP there are two drug-target interactions. One is an interaction between hsa:1584 and Spironolactone, while the other is hsa:2230 as a target for Flavin adenine dinucleotide. The importance of these relations for ACC is that hsa:1584 and hsa:2230 are the only known targets for the only accepted drug for ACC (mitotane). The therapeutic effect of mitotane is caused by its influence on these targets, which is documented in DrugBank (http://drugbank.ca). It is notable that these drugs are predicted from among 11140 possible drugs (i.e. the total number of drugs in the input dataset).

We explore these predictions by analyzing data from DrugBank (DB) and SuperTarget. Through interactive searching using the DB online interface, we are able to access additional details that were not included in the downloaded data used as input data for the predictions in Table 1. For example, the interactive searching identified 15 different drugs associated with target hsa:1584, including Spironolactone. In the downloadable files provided by DB (which are used in input data) only mitotane and metyrapone are related to hsa:1584 (the difference in data available by download vs. interactive online searching may be related to certainty, where only highly specific relations are listed in the downloadable files). SuperTarget also contains several drugs for hsa:1584, including Spironolactone (Hydrochlorothiazide). This analysis serves to validate the novel predictions made here and indicate that Spironolactone could be a good candidate for a novel therapeutic approach to ACC.

The second predicted relation (hsa:2230 and Flavin adenine dinucleotide), does not appear to be discussed in the literature but could be a good candidate for subsequent experimental analysis as a novel ACC therapy.

### Drug-disease relations

The only reported candidate for ACC in our input datasets is mitotane. In addition to mitotane, Heter-LP predicted one other drug candidate for adrenocortical carcinoma, cosyntropin. Cosyntropin is one part of the natural corticotropin hormone (ACTH). This hormone stimulates the adrenal cortex, which results in the production and secretion of adrenocortical hormones. In fact, cosyntropin produces cortisol and adrenaline by stimulating the adrenal glands and has traditionally only been used to diagnose and resolve adrenal gland problems (e.g., Addison’s disease, adrenal insufficiency caused by the use of steroids or from tumors).

On the other hand, mitotane, which is the first known therapeutic option for ACC patients, causes adrenal insufficiency and, due to its adverse effects on adrenocortical cells, often requires hydrocortisone replacement therapy (https://clinicaltrials.gov/). It is therefore hypothesized that cosyntropin can be used as an adjunct to mitotane to re-stimulate the adrenal glands. This promising prediction is achieved from among the 11140 different possible drugs.

### Disease-target relations

As explained in the introduction, targeted therapy is an effective therapeutic approach for treating various types of cancers. The principal goal is to find proteins that are critical to the disease process. In this way, one can target the proteins to return the status to reasonable conditions. Heter-LP can be used to predict novel targets for a given disease. We here examine the five novel gene and protein targets predicted by Heter-LP to be associated with ACC disease (see Table 1). These predictions are achieved from among 5568 possible targets.

- hsa:3480 (IGF-1R): As explained in the targeted therapy section, IGF-1R inhibitors are currently being considered for ACC. Although their use in the treatment of ACC is not yet widely adopted in clinical practice, it is an essential consideration in clinical trials. This relation is not explicitly included in the input data; the ability of Heter-LP to predict this relationship is notable.
- hsa:7153 (TOP2A): Jain *et al*.^31^ examined the expression and function of Top2A in human adrenal gland diseases. Their main propose was to examine the anti-ACC properties of factors that target Top2A. It has been shown that the expression of this gene in ACC is much higher than normal and benign tumors, which, in turn, is higher than observed in normal tissue. They went on to examine TOP2A inhibitors and demonstrate that TOP2A inhibitors show good anticancer activity against ACC. Finally, they conclude that TOP2A inhibitors are effective in controlling ACC metastasis and should be tested clinically. The fact that Heter-LP discovered this link as a novel predicted relation illustrates its ability to predict novel and viable hypotheses worthy of further investigation.
- hsa:1717 (DHCR7): According to the ctdbase (http://ctdbase.org/detail.go;jsessionid= 646E5B51C39B893F65017843961E9D37?type=gene&acc=1717&view=disease), ACC is one of the diseases associated with hsa:1717. GenomeNet (http://www.genome.jp/db/pcidb/ kna_cpds/23747) also indicates that hsa:1717 is the reductase enzyme for “7-Dehydrocholesterol / Provitamin D3, Cholesta-5,7-dien-3beta-ol”, a compound known to be associated with adrenal carcinoma. The relation between Adrenocortical carcinoma and DHCR7 is also discussed in^32^. These resources, that were not used by Heter-LP in making this prediction, serve to reinforce our confidence in both Heter-LP and its novel predicted relationships.
- hsa:4157 (MC1R): MRAP (Melanocortin Receptor Accessory Protein) is a protein that has a significant effect on the expression and function of MCR. In the adrenal gland, MRAP is an essential factor in the expression of MC2R / ACTH receptor functional expression. MC2R and MRAP are mainly involved in the adrenal cortex, and it has been proven that patients with cortisol deficiency have mutant lesions in MC2R and MRAP [11]. A previous study [11] describes the relationship between the MCR family, especially MC2R, with the adrenal glands and their function. Several studies have examined the role of MC1R in adrenal insufficiency [12, 13]. This novel prediction of Heter-LP is verified in this way.
- hsa:4215 (MAP3K3): According to our studies, no direct relationship is reported between hsa:4215 and ACC. However, GeneCard (http://www.genecards.org/cgi-bin/carddisp.pl?gene=MAP3K3) mentions that one of the phenotypic series related to hsa:4215 is endocrine/exocrine gland phenotype group. ACC and its related phenotypes are members of this group. Therefore, this novel prediction is also plausible and warrants further investigation.

Based on these results, it can be concluded that Heter-LP has been successful in predicting plausible and interesting disease-target relationships. These predictions present novel testable hypotheses for subsequent investigation by researchers in the pharmaceutical and medical sciences.

### Predicted relations by DT-Hybrid

As mentioned in Table 2, DT-Hybrid predicted only two novel targets associated with ACC: hsa:6409 (SEN2) and hsa:595 (CCND1); no other relations were predicted by DT-Hybrid for ACC and there is no overlap between the predictions of DT-Hybrid and Heter-LP. A literature search was conducted to verify the DT-Hybrid predictions. SEN2 could not be verified, and no published relation with ACC could be identified. However, CCND1 has been suggested as a molecular marker of cell cycles that may regulate gene expression related to ACC^33,34^.

As a second attempt to validate the DT-Hybrid predictions, we first searched for Adrenocortical Carcinoma in the disease section of KEGG (Kyoto Encyclopedia of Genes and Genomes -http://www.kegg.jp), producing known associated drugs, pathways and related genes. This search resulted in the following known AAC-related targets: ACTH-R (deletion) [HSA:4158], GNAI2 (mutation) [HSA:2771], N-ras (mutation) [HSA:4893], IGF II (overexpression) [HSA:3481], p53 (LOH, mutation) [HSA:7157], p16/INK4A (LOH, low expression) [HSA:1028], and MEN1 (LOH, mutation) [HSA:4221]. Note that there are established relationships between some of these known AAC targets and those predicted by Heter-LP, as discussed in section 2.3 and in section 8.2 of the supplementary file. However, there is no relation between these genes, and the two DT-Hybrid predicted targets. Therefore, we tried to investigate these predicted targets by searching KEGG for the genes SEN2 and CCND1. SEN2 had no results, while CCND1 is only known to be associated with non-AAC cancers such as oral cancer, esophageal cancer, breast cancer, and laryngeal cancer.

## Materials and methods

### Data preparation

Here, as mentioned before, the heterogeneous network consists of three sub-networks, drugs, diseases, and targets. Six separate matrices are required: (1) drug similarities, (2) disease similarities, (3) target similarities, (4) drug-disease relations, (5) disease-target relations, and (6) drug-disease relations. Leveraging several data sources, we gathered data for each element of the Heter-LP model and organized them as a comprehensive dataset that is available through GitHub (https://github.com/dkrlab) and the DKR site (http://dkr.iut.ac.ir/projects) (a detailed description of this dataset is also presented with it). In total, we gathered a collection comprising 11140 drugs, 11494 diseases, and 5568 targets. A brief description of using data in addition to previously known relationships involving ACC is also available in the supplementary file.

### Methods

After gathering the necessary datasets described in Section 3 and the construction of our network model, Heter-LP was run to discover drug-target, drug-disease, and disease-target links relating to adrenocortical carcinoma. Pseudocode and the workflow of Heter-LP is presented in detail in our previous paper [7] and is also included with the Heter-LP source code in GitHub and the DKR website (within the same project as the datasets mentioned in Section 3.1).

To provide the opportunity to compare the results of Heter-LP with other drug repositioning methods, we also apply DT-Hybrid to our input data. It is necessary to mention that DT-Hybrid can only be run on a justified data set, such that item names in different sub-networks are all consistent. For example, drugs listed in the drug-drug subnetwork should be shared with drugs listed in the two other sub-networks related to drugs (drug-disease and drug-target); likewise for targets and diseases. Therefore, we had to find a common list for each item and customize the input dataset such that DT-Hybrid is only applicable to 313 common diseases, 1403 common drugs, and 738 targets. A detailed explanation of prediction results for both Heter-LP and DT-Hybrid is presented in the next section.

## Conclusions

While the pharmaceutical industry has explored the use of drug repositioning in the field of rare diseases, this work has been hampered by a lack of a fundamental and systematic approach. Fortunately, in recent years, the necessity of extending the bioinformatics methods in this area has been recognized. In this regard, our team has proposed a hybrid computational approach based on the integration of biological networks for drug repositioning. We have here demonstrated the utility of Heter-LP, our method of label propagation in heterogeneous networks, to discover new drug repositioning leads for Adrenocortical carcinoma. In the first stage, we collected and compiled the necessary data for the diseases and genes concerned, the drugs, and their targets. These data were used in the second stage to predict novel drug-disease, disease-target, and drug-target relationships about ACC, a rare disease. The plausibility of several predicted links was supported by literature and datasets not used in the actual prediction of the links. It is necessary to mention that not one of the available computational drug repositioning methods has demonstrated such success as Heter-LP in suggesting novel therapeutic avenues for rare diseases.

In drug development, one of the critical points is the study of the interactions of a drug with its targets to determine the therapeutic effect of that drug on various diseases. On the other hand, in new treatments (especially the treatment of various types of cancers), the relationship between a disease and key proteins within the disease process has become more prominent, and this targeted therapy approach has shown good promise. Therefore, in addition to the use of well-known therapeutic drug-disease relationships, we have also used drug-target interactions and disease-protein/gene relationships to provide a list of potential candidates for repositioned drugs for ACC. According to the results and studies carried out, Heter-LP has been able to provide appropriate predictions for all three types of relationships. In some cases, there is a substantial supporting data to confirm these relationships and, although these relationships have not been recorded in the latest available datasets, they are being pursued by medical researchers with some entering the final evaluation process. These examples serve to validate the application of Heter-LP to drug repositioning for rare diseases.

### Data availability

Freely available under the IUT license at https://github.com/dkrlab and http://dkr.iut.ac.ir/projects

## Supplementary information

Supplementary file.
