# Supplementary material for: A computational drug repositioning method applied to rare diseases: Adrenocortical carcinoma
Source: Sci Rep. 2020 Jun 1;10:8846. doi: 10.1038/s41598-020-65658-x (PMC7264316; doi:10.1038/s41598-020-65658-x)
Supplement: Supplementary file 1 — Supplementary file. [file 41598_2020_65658_MOESM1_ESM.docx]

Supplementary file of

A computational drug repositioning method applied to rare diseases: Adrenocortical carcinoma

Maryam Lotfi Shahreza, Nasser Ghadiri, James R. Green

# Adrenocortical Carcinoma (ACC)

ACC affects an average of 0.72 cases per million people each year ^1^. The progression of this disease is rapid, generally progressing from the first observable clinical changes to advanced Cushing's syndrome in less than 12 months ^2^. Understanding the pathology of this disease, especially at the molecular level, is complicated due to the rare nature of the disease, and its treatment is not standardized due to the inability to conduct large-scale randomized trials. Approximately 40% to 60% of patients exhibit excessive hormone secretion, mostly due to excessive glucocorticoids, mineralocorticoids, androgens ^3^. Patterns of hormonal secretion can vary according to size, differentiation, and stage of tumor growth [4]. However, in approximately 30% of cases, the disease is not detected by sudden changes in hormones but rather due to the growth of the patient's tumor presenting as abdominal discomfort, back pain, and nausea or vomiting. In 10 to 15% of cases, the diagnosis of the disease arises indirectly without directly related symptoms ^3^; however, due to defective steroidogenesis within the tumor, the condition is often detectable due to severe hormonal changes ^2^.

# Therapeutic approaches for ACC

Mitotane cannot be given in substantial doses due to its toxicity; therefore simultaneous additional cytotoxic agents are typically employed. The cytotoxic agent most commonly used in the disease is cisplatin. Cisplatin in combination with mitotane provides a response rate of about 30%. The use of a combination of cisplatin with other cytotoxic agents, such as etoposide, doxorubicin, and 5-ﬂuorouracil, has not been able to improve this response rate in ACC significantly. Peixoto et al. ^4^ reported that oral metformin was useful in one case of metastatic ACC. Other medications can be used to reduce the production or effect of hormones resulting from this cancer ^1,5^. For example, ketoconazole and metyrapone can reduce the production of adrenal steroid hormones that can help alleviate the symptoms of this hormone but do not reduce the amount of cancer. Other anti-hormone drugs block the effects of hormones made by ACC tumors, including spironolactone, which decreases the effects of aldosterone; mifepristone, which reduces the effects of cortisol; and tamoxifen, toremifene, and fulvestrant that stop estrogenic effects (<https://www.cancer.org/cancer/adrenal-cancer>). One can refer to ^6^ for more information on the treatment options available for this disease.

In summary, although some research suggests that patients who have not taken mitotane after surgery are more likely to have a recurrence of ACC, many clinicians do not recommend mitotane since, in 80% of cases, severe side-effects can occur, often leading to discontinuation of therapy ^2^. Mitotane is a highly suboptimal solution, given its high toxicity in comparison with its therapeutic effect. Due to the rareness of ACC and the lack of economic benefits for pharmaceutical companies to design and develop new drugs for the disease, ACC drug repositioning studies are particularly important.

# The most promising protein targets for ACC

- Insulin growth factor (IGF-1,2): Changes in the expression of IGF-1,2 are often observed in ACC. Although IGF-1R inhibitors are not yet recommended for patients with advanced ACC. Several other growth factors are impacted by ACC, including epidermal growth factor (EGF), conversion factor α (TGF-α), and vascular endothelial growth factor (VEGF). Many receptors of these ligands belong to families of tyrosine kinase receptors whose inhibitors are currently used clinically in other malignancies ^2,5^.
- mTOR: Due to its association with the IGF-1R, mTOR inhibition is considered as a putative target for the treatment of ACC. It has been shown that inhibition of the mTOR signal decreases the growth of adrenocortical tumors in a mouse model. Studies involving mTOR are ongoing ^2,7^.
- Steroidogenic factor 1 (SF-1): Overexpression of SF-1 has been observed in ACC patients (especially children). It has been shown that inhibition of this factor inhibits the proliferation of ACC cancer cells ^2,7^.
- CTNNB1: Mutation of CTNNB1, the gene associated with β-catenin encoding, has been associated with ACC and adrenocortical adenomas ^2^. Because of CTNNB1 is frequently mutated in ACC, Hui *et al.* ^8^ have suggested that Nutlin-3a could be an effective agent for ACC. Nutlin-3a is a classic MDM2 inhibitor with an effective influence on CTNNB1-mutated Cancer Cells so could inhibit the growth of ACC cell with CTNNB1 mutation.
- Other putative factors: A number of other factors have been identified as possible targets for treating ACC, including VEGFR, PDGFR, KIT, FGFRs, RAF, PD-1, EGFR ^2,5^.

# Molecular events and signaling pathways related to ACC

Various researchers have attempted to identify genes, molecular structures, and pathways effective in adrenal cancer and have made good progress. Here we present some of the most important of these studies.

Szyszka et al. ^9^ reviewed the molecular alterations related to adrenocortical carcinogenesis. They also have attempted to describe the interactions between the involved genes, proteins and signaling pathways. According to Szyszka et al. ^9^, IGF2 (insulin-like growth factor 2) overexpression plays crucial roles in the formation of malignant adrenocortical lesions among the most frequent molecular events and IGF2 overexpression is observed in over 80% of ACC cases. IGF signaling is an important mechanism that take part in critical cellular processes including cell proliferation and survival. The IGF system in Homo sapiens is comprised of the two ligands IGF1 and IGF2, the insulin receptor (INSR), IGF1 receptor (IGF1R), IGF2 receptor (IGF2R), IGF-binding proteins (IGFBPs) and tyrosine kinase receptors. Szyszka et al. ^9^ have described that: "IGF2 exerts its function through binding to the membrane tyrosine kinase receptor (TKR), IGF1R. After the ligand is bound, the receptor autophoshorylates and the insulin receptor substrate 1 (IRS-1) is recruited. Tyrosine phosphorylation of IRS-1 activates the downstream signaling of pathways, such as PI3K/Akt/mTOR and Ras/Raf/ MEK/ERK." ^9^

Xiao et al. ^10^ have identified five genes (TOP2A, NDC80, CEP55, CDKN3, CDK1) that are significantly overexpressed in ACC. They also reported that the translational level of TOP2A (topoisomerase II alpha) was significantly higher in tumor tissues than in normal tissues. Their study is based on GEO datasets and mRNA biomarkers for ACC.

Gao et al. ^11^ have recently analyzed differentially expressed genes from adrenal tumor samples collected from patients undergoing adrenalectomy surgery. They identified three novel genes that appear to have a role in carcinogenesis: nuclear division cycle 80, cyclin B2, and topoisomerase 2-α. Furthermore, the authors reported that these three genes can predict overall survival and recurrence among the patients in the Cancer Genome Atlas database.

Adrenocorticotrophin (ACTH) is the principle regulatory hormone controlling steroid synthesis and secretion by adrenocortical cells [12]. Latronico investigated the behavior of adrenocortical cells and described the function of ACTH as follows: “The actions of ACTH are mediated by its specific membrane receptor (ACTH-R). The ACTH-R belongs to a subfamily of the G protein-coupled receptor superfamily, the melanocortin receptor family, which consists of the ACTH receptor (or MCR2), MSH-R (or MCR1) and three other receptors (MCR-3 to 5). Compared to normal adrenal tissue, ACTH-R mRNA expression was low in non-functional adenomas and carcinomas, intermediate in adrenocortical hyperplasias and cortisol-producing adenomas and high in aldosteronomas." ^12^

This behavior of ACTH-R gene in a subset of adrenocortical tumors suggest that the ACTH-R gene (and others in its family) can be involved in adrenocortical tumorigenesis, contributing to cellular dedifferentiation and malignant phenotype.

Those interested in further reading can refer to ^9-15^.

# Related works

## 5.1 Clinical drug repositioning approaches for ACC

Little research has examined the possibility of drug repositioning for treating ACC; furthermore, these few studies have employed only clinical and experimental approaches. One such analysis is the work done by Pantziarka et al. ^16^, which examined the therapeutic effects of mebendazole (MBZ) on various cancers, including ACC. This research is based on two clinical studies of the ability of MBZ to stop kidney cancer growth in mice and the effect of this drug on metastatic adrenocortical cancer in humans. According to the report, MBZ initially stabilized ACC patients without any adverse effects; however, it was ultimately not effective in treating ACC ^16^.

Another critical research on indications associated with ACC was done by Nilubol *et al*. ^17^. This study reviewed and evaluated drugs with antineoplastic effects on ACC, using a quantitative high-throughput drug screening (qHTS) method, including bortezomib, ouabain, methotrexate, and pyrimethamine. qHTS methods are based on the differential gene expressions and have been demonstrated to be effective in identifying drugs affecting cancer cells. However, there are two main problems with these methods: 1) their inability to detect drugs with 48 hours' delay in their effect, and 2) for most diseases, including rare diseases, cell lines are insufficient and are not accessible appropriately. Although the method described in ^17^ is an instance of a successful drug repositioning study for ACC, it was limited in the number of drugs that could be reasonably investigated. This inability to scale is due to the clinical nature of the methods employed and the lack of adequate cell lines. Such wet-lab screening methods require an initial list of the most probable candidates, which is the output of our proposed method, Heter-LP.

In another clinical drug repositioning study related to ACC, Satoh *et al.* applied qHTS methods to cell lines to examine 4,292 different drug compounds ^18^. Although initially sought to treat tapeworm infections in humans, niclosamide was shown to have anticancer activity in ACC through the weakening of WNT/β-catenin pathway (A cellular pathway with alternation in over 30% of ACC cases) and altered cellular metabolism ^18^. Niclosamide was shown to stop the growth of ACC in cancer cells in mice without causing significant side effects. Based on this research, niclosamide can now be used as a therapeutic and inhibitor drug for ACC. The most significant benefit of using this drug is its low toxicity, even in long-term use.

Ahmed et al. ^19^ examined the possibility of drug repositioning in WNT-dependent cancers, including ACC. They used a purely clinical approach in which 16 different drugs were identified as being effective in controlling WNT pathways. Niclosamide is one of the antiparalytic drugs whose anti-cancer indications were explained by this study.

Most research on ACC drug treatment consists of clinical case reports. For example, Lee *et al.* developed a case report that examined the effect of sunitinib on metastatic ACC ^20^. According to the report, sunitinib provides a relative treatment for about 7.5 months. In another case study, prepared by Fassnacht *et al*. ^21^, the therapeutic effects of four drugs, including mitotane, etoposide, doxorubicin and cisplatin (EDP plus Mitotane), were studied on 304 ACC patients over a period of five years.

Despite the efforts of various organizations, such as the European Network for the Study of Adrenal Tumors (ENSAT), in treating ACC and improving livelihoods of these patients, the disease is still largely uncontrollable, and practical treatment strategies remain elusive ^6^.

## 5.2 Computational drug repositioning approaches for rare diseases

Today, there is an ever-growing amount of biological data with the potential to benefit many application areas. The use of purely clinical and traditional drug repositioning methods are not able to scale and leverage these growing data. Accurate and efficient computational methods are required to fully utilize available data to present the most promising leads for consideration by biological scientists and to extricate researchers from confusion due to a large amount of data. Over the years, different methods for computational drug repositioning and drug-target interaction prediction have been presented, of which only a small number have been explicitly applied to rare diseases.

In one study ^22^, Sardana *et al.* emphasize the need to use drug repositioning for the treatment of rare diseases. In this study, the authors introduce and categorize the essential computational methods of drug repositioning. They also present some examples of cases where a drug associated with a non-rare disease has been successful in treating a rare disease, or vice versa. In another similar work Muthyala *et al.* ^23^, in addition to presenting various examples, have classified multiple strategies for the repositioning of drugs in rare diseases. In both studies, none of the introduced methods were applied for ACC. Most reported applications of drug reposition for rare diseases are for specific diseases, not general methods.

Nony *et al.* ^24^ have tried to provide a general framework for the development of rare diseases drugs through *in silico* model and experimental simulation techniques for rare diseases, using statistical analysis of existing clinical databases. In this framework, one first gathers epidemiological and randomized controlled trial (RCT) datasets, then tries to identify biomarkers related to drugs and disease to make a model for simulation of different options, and finally detects the most promising drugs. This framework may not be applicable for a disease such as ACC due to a lack of initially required datasets.

Another computational approach to develop drugs of rare diseases is HealNet, developed by Healx Solutions (https://healx.io/). It is claimed that HealNet will combine biological data analysis with machine learning methods to discover drug and disease relationships. The two main methodologies employed by Healx Solutions are drug repositioning and drug response prediction. The purpose of Drug Response Prediction is to predict how an individual patient will respond to a drug. This commercial system is not available for public use and therefore could not be directly compared with Heter-LP here.

# Data preparation

Here, as mentioned before, the heterogeneous network consists of three sub-networks, drugs, diseases, and targets. Six separate matrices are required: (1) drug similarities, (2) disease similarities, (3) target similarities, (4) drug-disease relations, (5) disease-target relations, and (6) drug-disease relations. Leveraging a number of data sources, we gathered data for each element of the Heter-LP model and organized them as a comprehensive dataset which is available through GitHub (https://github.com/dkrlab) and the DKR site (http://dkr.iut.ac.ir/projects), (a detailed description of this dataset is also presented with it). As a brief description:

- Three different sources of drug similarity: Chemical substructure similarities, side effect similarities, Anatomical Therapeutic Chemical (ATC) code similarities. The integration of these resources provides the similarities of 5089 drugs.

- Four different sources of disease similarities: Phenotype semantic similarities of OMIM (Online Mendelian Inheritance in Man (http://www.omim.org/)), disease genes similarities, similarity based on ICD-10 (International Statistical Classification of Diseases and Related Health Problems-10) classification, and semantic similarity based on DO (Disease Ontology). Similarities of 9886 diseases are provided by the integration of these resources.

- Four different similarities for protein targets: semantic similarities based on GO (Gene Ontology (http://www.geneontology.org/)), semantic similarities based on HPO (Human Phenotype Ontology (http://human-phenotype-ontology.github.io/)), semantic similarities based on DO, similarities based on KEGG (Kyoto Encyclopedia of Genes and Genomes ((http://www.kegg.jp)) pathway classification. The integration of these resources provides the similarities of 2940 targets.

- Two sources for drug-disease relationships: TTD (Therapeutic Target Database (<http://bidd.nus.edu.sg/group/cjttd/>)) and KEGG. Relations between 7382 drugs and 1970 diseases are provided by the integration of these resources.

- Two data sources for relationships between diseases and targets: KEGG and DiGeNet (http://www.disgenet.org). Relations between 1838 diseases and 4066 targets are provided by the integration of these resources.

- Two data sources for the relationship between drugs and targets: KEGG and DrugBank (http://drugbank.ca). Relations between 3350 drugs and 1415 targets are provided by the integration of these resources.

In total, we gathered a collection comprising 11140 drugs, 11494 diseases, and 5568 targets. The previously known relationships involving ACC within the input data are:

- Related targets: HSA:1718, HSA:1028, HSA:3623, HSA:2771, HSA:4221, HSA:3481, HSA:581, HSA:4158, HSA:6240, HSA:4221, HSA:7015, HSA:4893, HSA:7099, HSA:7157, HSA:9768.
- Related drugs (only approved): Mitotane, Tariquidar.
- Related diseases (similarity >= 0.3): adrenal cortical adenocarcinoma, neuroendocrine tumor, adrenal cortex cancer, pineal gland cancer, adrenal gland cancer, pituitary cancer, endocrine gland cancer, adrenal gland pheochromocytoma, adrenal gland ganglioneuroblastoma, adrenal medulla carcinoma, adrenal medulla cancer, extra-adrenal pheochromocytoma, adrenal neuroblastoma.

One problem that arises when examining diseases is the use of multiple names and sometimes identifiers for the same disease. For example, in the input data used in the present study, the following names have been used for adrenocortical cancer: adrenal cancer, adrenal carcinoma, adrenal gland cancer, adrenal gland carcinoma, adrenal cortical cancer, adrenal cortex cancer, and adrenocortical cancer**.** Furthermore, for some specific sub-types of the disease, there are also distinct and sometimes different names; for example adrenal cortical insufficiency, Addison's disease, hypocortisolism, chronic adrenal insufficiency, adrenal gland hypofunction, and adrenal cortical hypofunction. However, these all fundamentally refer to the same adrenal-related disease.

The use of different names in disease-disease, drug-disease and disease-target matrices compounds this problem. This study examines only one of the hundreds of diseases that are present and processed in our input data. Attempts were made to resolve these naming ambiguities through both manual and algorithmic approaches; however, the solution remains incomplete. The correction of these names requires close collaboration with the relevant disease-centric experts and the provision of strong standards. Some work has been done in this direction, including the provision of classifications such as the ICD-10 and the development of ontologies to capture semantic relations (including those used here: HPO, DO). Although we have used the latest and most authoritative information sources and data in this area, the problem remains. To ensure that the data quality relating to ACC remains representative of other diseases within our data, we did not perform any individual preprocessing for ACC and its different available names in the input data.

# Results

## 7.1 The complete list of novel predictions

The complete list of novel predictions associated with ACC, results for Heter-LP are presented in Table 1, Figure 1 (predicted drugs) and Table 2, Figure 2 (predicted targets), sorted according to their weights. Predicted weights are equal to computed probability for existence of the relation. The most important items are bolded (according to their predicted weights) and discussed in the main text.

Table 1 Predicted drugs for ACC by Heter-LP

| NO. | Drug | Predicted weight |
| --- | --- | --- |
| 1 | **Cosyntropin** | **0.02375** |
| 2 | Calcitriol | 0.0044375 |
| 3 | Tocilizumab | 0.004130125 |
| 4 | Anakinra | 0.00296875 |
| 5 | Tacrolimus | 0.002500875 |
| 6 | Cyclosporine | 0.00206625 |
| 7 | Prednisolone | 0.0013775 |
| 8 | Methotrexate | 0.0011875 |
| 9 | Citalopram | 7.61E-07 |
| 10 | Isoxsuprine | 7.35E-07 |
| 11 | Isoxsuprine | 6.03E-07 |
| 12 | Citalopram | 5.37E-07 |

Figure 1Predicted drugs for ACC by Heter-LP

Table 2 Predicted targets for ACC by Heter-LP

| NO. | Target | Predicted weight |
| --- | --- | --- |
| 1 | **HSA:3480** | **0.00678775** |
| 2 | **HSA:4157** | **0.00678775** |
| 3 | **HSA:4215** | **0.00678775** |
| 4 | **HSA:7153** | **0.00678775** |
| 5 | **HSA:1717** | **0.0059375** |
| 6 | HSA:2779 | 0.002158875 |
| 7 | HSA:1811 | 0.0013205 |
| 8 | HSA:6231 | 8.48E-05 |
| 9 | HSA:29851 | 1.08E-06 |
| 10 | HSA:3977 | 4.01E-07 |
| 11 | HSA:6005 | 2.49E-07 |
| 12 | HSA:26058 | 2.97E-08 |

Figure 2 Predicted targets for ACC by Heter-LP

## 7.2 Evaluation of results based on signaling pathways

Below, we provide some evidence for the efficiency of the five top predicted protein targets based on cellular processes of ACC.

- The top predicted target is HSA:3480 (IGF1R). As discussed in section 4, some studies have shown that insulin receptors (IRs), including insulin-like growth factor (IGF) 1 receptor (IGF1R), are important influencing factors in diseases that are involved in IGF2 alteration. Vaquero et al. ^25^ have found that, in Cholangiocarcinoma, an increase in IGF2 expression occurs with an upregulation of insulin receptor (IR) and that the insulin-like growth factor 1 receptor (IGF1R) is up regulated in resistant cells. IR/IGF1R inhibition reduced epithelial-mesenchymal transition (EMT) and a cancer stem cell (CSC)-like traits in resistant cells. Andersson et al. ^26^ have discussed the role of IGF signaling in some different carcinoma with a focus on the impact of IGF1R, for example in Adenoid cystic carcinoma (a clinically challenging tumor with a high rate of recurrence and distant metastases). According to their finding, IGF2 is the endogenous factor regulating IGF signaling in this disease and pharmacologic inhibition of IGF1R could reverse the oncogenic transcriptional program induced by overexpression of IGF2 in affected cells. As a result, they concluded that the IGF2 and IGF1R are two important axes for therapy in Adenoid cystic carcinoma. They described different situations that provide new important insights into the role of IGF1R signaling in fusion gene driven malignancies ^26^.

Szyszka ^9^ has described that: "Overexpression of *IGF1R* is also thought to play a role in the pathogenesis of ACC, but it seems to be primarily an indicator for paediatric ACCs. Promising pre-clinical data of anti-proliferative effects of IGF1R inhibition in ACC provided the basis for phase I trials of IGF1R inhibitors. The anti-IGF1R monoclonal antibody figitumumab has demonstrated biological activity in refractory ACC."

It is suggested that similar approaches to IGF1R may reduce cancer tumor growth by up to 76% [12]. Based on these results, Andersson and his colleagues believe that: "Future studies need to focus on evaluating IGF1R inhibitors in combination therapies or as an adjunct to conventional chemotherapy and to find new biomarkers that can identify patients who will benefit from IGF1R inhibition". ^26^

Thus we believe IGF1R warrants additional attention in understanding and treating ACC.

- The second predicted target is HSA:4157 (MC1R). According to section 4, MC1R could be used for mediation of ACTH (one of the major regulatory hormones associated with adrenocortical cells) ^12^.
- HSA:4215 (MAP3K3) is the third prediction. It is a member of Mitogen-activated [protein kinases](https://www.sciencedirect.com/topics/medicine-and-dentistry/protein-kinase) (MAPKs) family. MAPKs are serine-threonine–directed kinases that could be activated by different stimuli, including hormones, growth factors, [neurotransmitters](https://www.sciencedirect.com/topics/medicine-and-dentistry/neurotransmitter), cytokines, cell stress, and ECM proteins. The main MAPKs role is regulation of some cellular processes, including [gene transcription](https://www.sciencedirect.com/topics/medicine-and-dentistry/genetic-transcription), protein translation, metabolism, and [cytoskeleton](https://www.sciencedirect.com/topics/medicine-and-dentistry/cytoskeleton) function. Thus, MAPKs are key factors in the control of cell growth, differentiation, and [apoptosis](https://www.sciencedirect.com/topics/medicine-and-dentistry/programmed-cell-death). MAPKs also play a key regulatory role in the production of pro-inflammatory cytokines and downstream signaling events leading to joint inflammation. ^27^
- According to recent studies, the cell cycle G1/S transition is overexpressed in ACC, and TOP2A (our 4^th^ ranked predicted protein) is one of the important proteins involved in it. As described in section 4, Xiao et al. ^10^ have reported significantly overexpression of TOP2A in ACC, and TOP2A is one of the three important genes identified by Gao et al. ^11^.
- The relation of our fifth predicted protein, HSA:1717 (DHCR7), to ACC is discussed in Section 2.3 of main manuscript.

# References

1 Berruti, A. *et al.* Etoposide, doxorubicin and cisplatin plus mitotane in the treatment of advanced adrenocortical carcinoma: a large prospective phase II trial. *Endocrine-related cancer* **12**, 657-666, doi:10.1677/erc.1.01025 (2005).

2 Lebastchi, A. H., Kunstman, J. W. & Carling, T. Adrenocortical Carcinoma: Current Therapeutic State-of-the-Art. *Journal of Oncology* **2012**, 11, doi:10.1155/2012/234726 (2012).

3 Libé, R. Adrenocortical carcinoma (ACC): diagnosis, prognosis, and treatment. *Frontiers in Cell and Developmental Biology* **3**, 45, doi:10.3389/fcell.2015.00045 (2015).

4 Peixoto, R. *et al.* Efficacy of oral metformin in a patient with metastatic adrenocortical carcinoma: Examination of mechanisms and therapeutic implications. *Rare Tumors* **10**, 2036361317749645, doi:10.1177/2036361317749645 (2018).

5 Cabezon-Gutierrez, L. *et al.* *Clinical Case of Metastatic Adrenocortical Carcinoma With Unusual Evolution: Review the Literature*. (2016).

6 Ronchi, C. L., Kroiss, M., Sbiera, S., Deutschbein, T. & Fassnacht, M. EJE PRIZE 2014: Current and evolving treatment options in adrenocortical carcinoma: where do we stand and where do we want to go? *European journal of endocrinology* **171**, R1-R11, doi:10.1530/eje-14-0273 (2014).

7 Maluf, D. F., de Oliveira, B. H. & Lalli, E. Therapy of adrenocortical cancer: present and future. *American Journal of Cancer Research* **1**, 222-232 (2011).

8 Hui, W. *et al.* Nutlin‐3a as a novel anticancer agent for adrenocortical carcinoma with CTNNB1 mutation. *Cancer Medicine* **7**, 1440-1449, doi:10.1002/cam4.1431 (2018).

9 Szyszka, P., Grossman, A. B., Diaz-Cano, S., Sworczak, K. & Dworakowska, D. Molecular pathways of human adrenocortical carcinoma - translating cell signalling knowledge into diagnostic and treatment options. *Endokrynol Pol* **67**, 427-450, doi:10.5603/EP.a2016.0054 (2016).

10 Xiao, H. *et al.* Identification of Five Genes as a Potential Biomarker for Predicting Progress and Prognosis in Adrenocortical Carcinoma. *J Cancer* **9**, 4484-4495, doi:10.7150/jca.26698 (2018).

11 Gao, Z. *et al.* Expression profiles analysis identifies the values of carcinogenesis and the prognostic prediction of three genes in adrenocortical carcinoma. *Oncol Rep* **41**, 2440-2452, doi:10.3892/or.2019.7021 (2019).

12 Latronico, A. C. Role of ACTH receptor in adrenocortical tumor formation. *Braz J Med Biol Res* **33**, 1249-1252, doi:10.1590/s0100-879x2000001000016 (2000).

13 Pereira, S. S. *et al.* MAPK/ERK pathway inhibition is a promising treatment target for adrenocortical tumors. *J Cell Biochem* **120**, 894-906, doi:10.1002/jcb.27451 (2019).

14 Nicolson, N. G., Man, J. & Carling, T. Advances in understanding the molecular underpinnings of adrenocortical tumors. *Curr Opin Oncol* **30**, 16-22, doi:10.1097/CCO.0000000000000415 (2018).

15 Alshabi, A. M., Vastrad, B., Shaikh, I. A. & Vastrad, C. Identification of important invasion and proliferation related genes in adrenocortical carcinoma. *Med Oncol* **36**, 73-73, doi:10.1007/s12032-019-1296-7 (2019).

16 Pantziarka, P., Bouche, G., Meheus, L., Sukhatme, V. & Sukhatme, V. P. Repurposing Drugs in Oncology (ReDO)—mebendazole as an anti-cancer agent. *ecancermedicalscience* **8**, 443, doi:10.3332/ecancer.2014.443 (2014).

17 Nilubol, N. *et al.* Four clinically utilized drugs were identified and validated for treatment of adrenocortical cancer using quantitative high-throughput screening. *Journal of Translational Medicine* **10**, 198-198, doi:10.1186/1479-5876-10-198 (2012).

18 Satoh, K. *et al.* Identification of Niclosamide as a Novel Anticancer Agent for Adrenocortical Carcinoma. *Clinical cancer research : an official journal of the American Association for Cancer Research* **22**, 3458-3466, doi:10.1158/1078-0432.CCR-15-2256 (2016).

19 Ahmed, K., Shaw, H. V., Koval, A. & Katanaev, V. L. A Second WNT for Old Drugs: Drug Repositioning against WNT-Dependent Cancers. *Cancers* **8**, 66, doi:10.3390/cancers8070066 (2016).

20 Lee, J.-O. *et al.* Metastatic Adrenocortical Carcinoma Treated with Sunitinib: A Case Report. *Japanese Journal of Clinical Oncology* **39**, 183-185, doi:10.1093/jjco/hyn146 (2009).

21 Fassnacht , M. *et al.* Combination Chemotherapy in Advanced Adrenocortical Carcinoma. *New England Journal of Medicine* **366**, 2189-2197, doi:10.1056/NEJMoa1200966 (2012).

22 Sardana, D. *et al.* Drug repositioning for orphan diseases. *Briefings in bioinformatics* **12**, 346-356, doi:10.1093/bib/bbr021 (2011).

23 Muthyala, R. Orphan/rare drug discovery through drug repositioning. *Drug Discovery Today: Therapeutic Strategies* **8**, 71-76, doi:<http://dx.doi.org/10.1016/j.ddstr.2011.10.003> (2011).

24 Nony, P. *et al.* A methodological framework for drug development in rare diseases. *Orphanet Journal of Rare Diseases* **9**, 164, doi:10.1186/s13023-014-0164-y (2014).

25 Vaquero, J. *et al.* The IGF2/IR/IGF1R Pathway in Tumor Cells and Myofibroblasts Mediates Resistance to EGFR Inhibition in Cholangiocarcinoma. *Clin Cancer Res* **24**, 4282-4296, doi:10.1158/1078-0432.CCR-17-3725 (2018).

26 Andersson, M. K., Åman, P. & Stenman, G. IGF2/IGF1R Signaling as a Therapeutic Target in MYB-Positive Adenoid Cystic Carcinomas and Other Fusion Gene-Driven Tumors. *Cells* **8**, 913, doi:10.3390/cells8080913 (2019).

27 Hellmich, M. R. & Evers, B. M. in *Physiology of the Gastrointestinal Tract (Fourth Edition)* (ed Leonard R. Johnson) 435-458 (Academic Press, 2006).
